# Tax Justice Beyond National Borders—International or Interpersonal?

**DOI:** 10.1093/ojls/gqab026

**Published:** 2021-08-11

**Authors:** Johanna Stark

**Keywords:** international taxation, international tax law, distributive justice, global justice, collective responsibility

## Abstract

Recent times have seen growing calls for considerations of justice to be given a greater role in international taxation. The main driver of these calls are distributive concerns, although agreement is still missing as to what this means both in principle and in practice. This article asks whether it is the task of international tax law at all to implement principles of distributive justice beyond the national context and gives an overview of how the ‘global justice debate’ in contemporary political philosophy bears on this question. When it comes to distributive duties with respect to taxing rights, it is crucial to differentiate between the collective and the individual level. Absent a robust assumption of a benevolent and capable government on the recipient side, the reallocation of taxing rights from state to state does not necessarily help when it comes to fulfilling duties of justice towards individuals.

## Introduction

There seems to be agreement that the international tax order needs profound reform, that the rules governing international taxation need to be adapted not in a piecemeal, but in a systemic fashion to increased levels of global economic integration, taxpayer as well as capital mobility and new types of business models. Various arguments have been brought forward regarding the principles that such reforms should follow, and the specific changes that are necessary to arrive at a new international tax order worthy of the name. Besides the common mantra that the international distribution of taxing rights should be (more) closely aligned with the creation of economic value, we increasingly hear calls that the international community should strive for ‘more justice’ in the international tax system, particularly with a view to the outcomes it produces for developing countries.^1^

Proponents of this approach often base their claim on drastic inequalities in the global distribution of resources and life chances, and on the role that international taxation could—and arguably should—play in remedying this situation by enabling a redistribution of resources towards less affluent parts of the world.^2^ Political philosophers, economists and political scientists have devoted increasing attention to the question of whether and why global inequality is a problem, who bears the responsibility for bringing about social and political change, and how this change might feasibly be achieved. In the domestic context, taxation is perceived as the state’s primary instrument for resource redistribution,^3^ even if the particular design and scope of such redistribution has always been the subject of considerable political dispute. Dealing with global poverty and inequality, however, has received far less attention in this tax justice debate.^4^

Strengthening considerations of justice in the set of objectives that guide international tax policy is a goal that leaves ample room for interpretation.^5^ This article is about one specific way of framing the issue of justice in international taxation: is there a convincing argument to be made that some states are, as a matter of *distributive justice*, under a duty to agree to a rearrangement of taxing rights^6^ that leads to a redistribution of such rights to other states? A critical discussion of the assumptions that need to be made in order to answer this question in the affirmative leads to the conclusion that some of these assumptions are hard to defend. Following the current, introductory section, section 2 seeks to establish conceptual clarity as to the distributive dimensions that play a role in this debate. Section 3 gives an overview of the philosophical ‘global justice debate’ that revolves around the meaning of distributive justice beyond the national context, and the duties it entails for moral agents in a world that sees increasing political, economic and cultural integration across national borders.^7^ The structural implications of this debate’s main theoretical branches for the characteristics of a just international order are briefly discussed in section 4. These implications are relevant for international tax law insofar as it is part of this international political and economic order, and significantly contributes to the allocation of advantages gained from global economic co-operation.^8^ Section 5 argues that we must differentiate between the individual and the collective level when making claims of justice and that, ultimately, duties *of distributive justice* with respect to a particular allocation of *taxing rights* between states are difficult to defend. Section 6 briefly sketches alternatives to conceptualising the need for reforms in international tax law as a matter of duties of distributive justice, before section 7 provides a conclusion.

## Distributive Dimensions of International Taxation

Considering the many ways in which distributive justice may be understood as a normative principle of international tax law, it may help to first differentiate between taxation as a political *instrument* for (re)distributing resources and taxes as the *object* of distribution in an international setting.^9^

### A. Distribution by Taxation

The instrumental dimension has so far been addressed mainly in the context of national tax justice, but in principle applies to the international or global level as well. We can discuss the equitable design of a distributive scheme in a meaningful way even if we do not define the group of relevant actors on both the revenue and expenditure sides by national affiliation, but rather by looking beyond national borders. With regard to the genuinely international context that involves taxes as the object of a distributive scheme, the debate’s focus has been on the international allocation of taxing rights pertaining to the profits of multinational enterprises. As a contribution to this debate, the following sections refer primarily to the taxation of multinational enterprises, although the philosophical arguments advanced here are not limited to this context. The distributional effects of a social scheme that involves the pooling of resources can be assessed only by looking at its overall consequences on both the revenue and the expenditure side, not through a short-sighted perspective on only one of the two sides, or on only one type of tax on the revenue side, such as the income tax.^10^ Nevertheless, a distinction must be made between the normative considerations that apply to the revenue side on the one hand and on the other hand those that guide policy with respect to the expenditure side. Social justice, according to Charles Beitz, has ‘two faces. One looks toward the distribution of the benefits of social life; the other, toward the allocation of its burdens.’^11^ Applied to the tax context, the issue on the revenue side is about who has to pay how much into a pool of resources. This is where the tax debate on horizontal and vertical equity comes in, the principle of efficiency versus equity and the demand that everyone must pay their ‘fair share’, a claim that is *prima facie* plausible but difficult to spell out in detail. On the expenditure side, the question is how the available resources and benefits are to be distributed. Criteria such as equality, individual merit or need may be considered as decisive factors here.^12^

A second differentiation that is worth mentioning here is the one between reasons that speak for or against the (international) redistribution of resources in general and the comparative advantages and disadvantages of several instrumental ways of going about such redistribution.^13^ Because ‘why’ and ‘how’ to redistribute require different kinds of answers, the conclusion that global inequality must, from a moral standpoint, be remedied does not necessarily mean that international tax law is the most appropriate instrument with which to achieve the task, or even an apt instrument at all. The ‘how’ dimension of international redistribution is of central importance when it comes to spelling out the ways in which positive answers to the ‘why’ question can be put into political and legal practice.^14^ There is much to be said about what an effective use of the international tax system for redistributive purposes would require (in terms of institutional reforms, for instance, or an overhaul of the current tax treaty network), but these aspects of the ‘how’ dimension are beyond the scope of this contribution,^15^ as is the comparative assessment of various other channels though which redistribution of resources across national borders could be achieved.

### B. Distribution of Taxing Rights

Normative questions of a different nature arise with respect to taxes not in their instrumental role as giving effect to (re)distributive policies, but as an object of distribution themselves.^16^ This perspective is central when it comes to the taxing rights of several political units with respect to the same tax base, particularly the international allocation of taxing rights with regard to the profits of multinational enterprises. Again, two questions must be distinguished: first, who can tax at all? And second, how are multiple taxing rights to be coordinated?

For the first question, international taxation currently relies on ‘economic allegiance’: profits generated across national borders are only subject to tax where a sufficiently robust economic connection can be established. However, the territorial nexus criterion offers no guidance on how to distribute the ‘tax cake’ among all those who have legitimate demands on a piece.^17^ This is where the debate on value creation comes in, which has recently gained prominence in the context of the OECD/G20’s base erosion and profit shifting (BEPS) initiative. This debate addresses the challenge of properly allocating the economic activities of multinational enterprises in order to align the international distribution of taxing rights with economic reality.^18^ So far, however, a coherent allocation system has not been achieved,^19^ the main reason being that the concept of value creation, as appealing it might seem at first sight, upon closer inspection fails to provide any guidance on how to deal with specific distributional conflicts.^20^ Clear answers can be derived only in a negative and therefore limited fashion, and only with respect to the first question above as to who can tax at all: regardless of the difficulties in finding a definition that yields clear answers as to where to locate which portion of the overall value that multinational enterprises create, a bare ‘paper presence’ in one of the world’s tax havens does not qualify.^21^

This perspective is structurally different from the view of taxes as a political instrument. The distributive scheme described above is a three-pole scenario: in addition to the revenue side and the expenditure, there is some form of superior authority that determines both the scope and mechanics of redistribution, and ideally ensures stable practical implementation. Here, however, we are dealing with a bipolar relationship: in essence, we are looking at states as actors who have to find agreement on a particular division of the ‘tax cake’ at hand.^22^

## Distributive Justice in Contemporary Political Theory

The opposing positions in the current philosophical debate about the correct understanding of justice in the political context differ significantly as to which relationships are governed by principles of justice at all.^23^ Do individuals solely qualify as parties of the relation? Does the relationship between institution and individual also qualify? Can duties of justice also exist between institutions or other collectives, such as states?^24^ A related question is whether inter-institutional justice, if conceptually possible, is guided by the same principles as it is on the interpersonal level. The term ‘international justice’ suggests an understanding that not only recognises states as relevant entities, but even places them at the centre of normative assessment.^25^ The term ‘global justice’, however, comes with a connotation of individuals as the main reference points.^26^

Positions on the theoretical spectrum of political justice can be further differentiated by the content that the ensuing duties are taken to have and the ideal of a just state on which they are based: for some, the overarching objective that is to be achieved by redistributing resources is ultimately some form of equality,^27^ whereas for others it is the satisfaction of some properly defined class of basic human needs.^28^

Based on these differentiations, the most important positions of the multi-faceted ‘global justice’ debate in political philosophy are very briefly presented below. The discussion is limited to the dimension of socio-economic distributive justice and its realisation through the redistribution of scarce resources in an international context.^29^ Important forks in the by now widely ramified landscape of theories can be illustrated by the following two questions: firstly, which structural conditions trigger distributive responsibilities? And secondly, are these conditions currently met in an international context?

The following sections are devoted to three groups of philosophical theories that provide different answers to the questions outlined above and have decisively shaped the discussion on the scope of distributive duties in recent decades. The starting point are forms of contemporary contractualism (A), followed by cosmopolitan approaches (B) and so-called political theories of justice (C).

### A. Contemporary Contractualism

Even the briefest description of contemporary theories of justice cannot ignore John Rawls’s *A Theory of Justice*.^30^ Rawls’s two principles of justice are a central building block of the ‘basic structure’, the institutional design of a society based on the idea that, from a moral point of view, accidental circumstances should not be the basis for a drastically unequal distribution of resources.^31^

Like many of its historical predecessors, the Rawlsian theory of justice is political, in that the frame of reference for the justice of a social order is always a kind of *polis*. Parties of the moral relationship to which principles of justice apply are primarily the state with its institutions on the one hand and its citizens on the other.^32^

The international order, as the term already suggests, is historically based on a network of interstate relationships as reflected by a web of international agreements. In a Rawlsian world, in addition to the domestic framework on the first level, we have the international community on the second level, with nation states as key actors. This two-level model is characterised by normative pluralism: the criteria for a good order in the national and international context are not identical; genuine principles of distributive justice are only applicable within the national framework. It is this normative pluralism that the cosmopolitan critique of Rawls’s theory is based upon.

### B. Cosmopolitan Theories of Justice

Early representatives of moral cosmopolitanism, such as Charles Beitz and Thomas Pogge, have argued for the application of the two principles of justice to the international context as well.^33^ The practical implications of such an extension are drastic: if an unequal distribution of resources can only be justified if—now seen globally—it benefits the least privileged, we face an obvious need for radical redistribution.

From a theoretical point of view, the critique is *prima facie* plausible: how can it be that the freedoms, rights and life chances of individuals are what create moral duties towards them, but at the same time the responsibilities to guarantee these freedoms and rights depend on the national affiliation of the individuals concerned? For Pogge, the nationality of a person is merely another morally irrelevant ‘deep contingency’,^34^ which *per se* cannot legitimise an unequal distribution of resources. The moral contingency of citizenship results, as Ayelet Shachar aptly describes it, from ‘archaic mechanisms of its attribution depending on the birth of a human being’.^35^ It is thus not surprising that the combination of the universalist justification of the difference principle and the limited scope of its application to the national context has been perceived by many as contradictory.^36^

Moderate cosmopolitans such as Beitz and—at least in his early writings—Pogge base their arguments on the central importance of a social ‘basic structure’ as a normative basis of distributive duties. They accept the premise that distributive duties according to the difference principle can only result from a sufficiently robust interactive relation between the parties involved, but deny that this relation can only exist within a state. Unlike Rawls, they regard such a basis as already existing beyond national borders. According to this line of argument, the relevant social context is the world society of all people that has formed in the course of global political and economic integration and exhibits a sufficient degree of robustness. The institutions of international trade in particular, such as the WTO, World Bank and OECD, are cited as signs of how advanced, diverse and stable co-operation relations and economic interdependencies across national borders have become.^37^ If state borders do not (any longer) reflect the limits of social and economic co-operation, so the argument goes, they should not be the decisive criterion for the scope of distributive duties.^38^

What these positions have in common is the premise that certain types of institutions or rule-based practices can trigger distributive duties among their participants.^39^ Belonging to a group entails mutual obligations; the strength and extent of these obligations depends on the group’s degree of integration. This explains our intuition that our duties to family members are different and stronger than to a person living far away and whom we have never met. Because the connection to institutions and rules leads to different configurations of justice obligations depending on the context, these conceptions are also called particularist. A decisive difference to the Rawlsian model is that duties of justice do not exist between institutions and their participants, but primarily between these participants themselves.

### C. Political Theories of Justice

Rawls himself expressly refused a cosmopolitan interpretation of his theory of justice.^40^ In the course of the debate initiated by Beitz and Pogge, Rawls and other representatives of a political understanding of distributive justice have attempted to explain why justice, especially distributive justice, can only be conceived as a value and a political ideal within a national framework. According to this understanding, the drastically unequal global distribution of resources is not a problem of distributive justice in the narrower sense.

Statist positions are theories according to which the state is a privileged forum as far as justice is concerned. Under the heading of a cultural or liberal nationalism, distributive duties are derived from a form of pre-political solidarity^41^ that builds upon common group membership. Proponents of a cultural nationalism see no contradiction in privileging the citizen relationship over universal human rights. Instead, in their view, it is a *non sequitur* to conclude from the existence of universal rights that the obligation to realise these rights falls on everyone’s shoulders in exactly the same way.^42^

Representatives of a cultural nationalism are not so much concerned with limiting the scope of application of distributive justice to the national as opposed to the global context. They do, however, run into problems distinguishing fellow citizenship from common membership in other, spatially and numerically far less comprehensive groups, such as religious communities, some of which exhibit a significantly higher degree of cultural integration and a stronger ‘sense of a “we” that shares a common fate’.^43^

A statist argument of a different kind corresponds well with Rawlsian principles of justice. Thomas Nagel has pointed out that such principles must not be viewed in isolation from their overall political function: in the end, they contribute to the justification of the state’s monopoly on the use of force.^44^ Nagel’s argument is not based on any kind of pre-political association, but on the characteristics of a constitutional state as a genuine prerequisite for duties of distributive justice.

Nagel spells out his position in two arguments. First, following Thomas Hobbes, he emphasises the connection between justice and sovereignty: duties of justice depend on an enforcement mechanism that only a sovereign can guarantee—without a legal system supported by a state monopoly of force, coordination of behaviour and stability of expectations within a large group of people cannot be achieved.^45^ Secondly, egalitarian justice is determined by the collective responsibility for the rules governing a state: their addressees are not only subjects, but at the same time authors of these rules. If we take this ‘co-authorship’ seriously, the claim to validity of state rules can only be legitimised by the fact that they can be justified equally towards all addressees concerned.

Nagel’s position deserves to be described as a political theory of justice in the narrower sense because it postulates not only any kind of institutional solidarity, but membership of a sovereign state as an indispensable prerequisite for duties of distributive justice. A ‘pre-institutional’ understanding of justice as a universal ideal of interpersonal relationships, as it is found at the core of radically cosmopolitan theories, is misguided. Against this background,^46^ the relationship created by social co-operation in the form of trade and exchange of goods cannot be compared with membership of the same state.^47^ A global sovereign, however, does not exist and will not come into existence in the foreseeable future. Nagel does not fundamentally deny the possibility of a global constitutional state in which a sufficiently robust regulatory power provides the basis for genuine sovereignty.^48^ He stresses, however, that the current situation does not give rise to such hopes.^49^ In this realism, but not in the explanatory pattern, he differs from global contractualists such as Pogge and Beitz.

At least for the time being, this means that without an international legal system with a quasi-state monopoly on the use of force outside individual states, there is no basis at all for the application of distributive justice as a political ideal.

## Distributive Justice and the Architecture of International Taxation

What does the philosophical discussion presented above mean for the legal rules that govern international taxation?^50^ What would it mean or what would it take to align the international tax framework with the implications of these theories? To what extent does the current state of affairs already conform to one theoretical approach or another?

The current architecture of international taxation is based on statist premises.^51^ If duties of justice are limited to the national domain, little thought has to be given to a fair structuring of the international tax landscape. It is therefore not surprising that cosmopolitan positions result in a more far-reaching need for reform than a concept of political justice already anchored in the current system.

One strategy for identifying reform options for the international tax system is purely instrumental and first involves the assessment of distributional outcomes that can in some way be causally linked to the international tax regime that is currently in place. Comparing this current state of affairs with a situation characterised by ideal distributive justice (for which there are, as seen above, several theoretical proposals), the variance between the two worlds demonstrates a concrete need for redistribution to the benefit of some at the expense of other people or political units—depending on who is regarded as the relevant moral subjects and thus party to the justice relation. Seen from this instrumental angle, it makes sense to inquire which particular ‘screws’ in the current system might need adjustment and in which direction in order to bring us closer to the ideal result.

A question of immense practical importance is whether the acceptance of some form of moral cosmopolitanism would entail a commitment to some form of legal cosmopolitanism as well. Does recognition of the global nature of our redistributive duties require a structural counterpart in the international legal order, in this case in international tax law? The answer to this question has far-reaching implications for who is responsible for managing mandated redistribution. In the statist picture, these are the nation states themselves; from a legal cosmopolitan perspective, a reallocation of taxing rights and administrative responsibilities to the supranational level is inevitable.

The following paragraphs deal with two different ways in which global justice in the sense of moderate cosmopolitanism could be achieved: first, ideas from the philosophical literature that go hand in hand with radical structural reforms, especially with regard to the allocation of taxing rights; and second, possibilities of realising global justice without fundamentally changing the current multi-level system of international tax law.

### A. Reform Proposals

In addition to the philosophical justification of universal duties of justice, Pogge has focused his work on practical strategies and political instruments that could be used to mitigate the drastically unequal distribution of resources and life chances across the globe that we see today.

#### (i) New tax bases

Pogge is not primarily concerned with international taxation; nevertheless, taxation in one form or another plays an important role in his and other cosmopolitans’ instrumental considerations. According to Pogge’s much debated proposal of a so-called ‘global commodity dividend’,^52^ states would not have unrestricted ownership of the resources located on their territory, but would have to pay a tax on the extraction and consumption of, for example, mineral resources.^53^ The idea behind this proposal is that the distribution of natural resources is another morally arbitrary ‘deep contingency’ that can legitimately be addressed and compensated by redistribution.

A similar proposal is a global health fund financed by wealthy countries, designed to increase incentives for pharmaceutical companies to develop medicines to combat poverty-related diseases.^54^ With some other proposals, the link between the choice of tax base and the redistributive purpose is less obvious, such as in the case of an ‘air ticket tax’ to finance global health programmes.^55^ The common goal of these proposals is to generate resource pools that can be utilised to meet the basic socio-economic needs of the world’s global poor.

Shachar derives an argument for a ‘birthright privilege levy’ from the morally arbitrary status of being a citizen of a socio-economically privileged state. In the context of current citizenship law, citizenship status is conceived analogously to an inherited bundle of rights, in particular in terms of access rights to resources and life chances.^56^ The idea of a birthright privilege levy is thus a kind of global inheritance tax on the intergenerational transfer of benefits associated with privileged citizenship.

Further ideas and proposals aim at the protection and financing of global public goods, such as climate and environmental protection as the objectives of a ‘carbon tax’ on the consumption of fossil energy sources and the emission of greenhouse gases. It is in this category that the long-debated financial transaction tax belongs,^57^ which is intended to curb the supposedly destabilising effects of, for example, short-term currency speculation on state budgets and real economies, especially in the developing world.^58^ Lesser-known examples include taxes on the sending of e-mails in industrialised countries to compensate for the ‘digital divide’ towards developing countries, on the sale of luxury goods or on the international sale of arms.^59^

#### (i) Globalisation of taxation

Inextricably linked with the debate on which particular tax bases could be used to add regulatory precision to the traditional *trias* of income, capital and consumption and to finance global redistributive schemes are the questions of how such schemes should be organised, who should be responsible for tax collection and who should be entitled to decide on the use of the funds thereby generated.^60^

From a cosmopolitan perspective, it is not only the result in terms of a certain distribution of relevant resources that matters, but also the path towards such a distribution. *Prima facie*, it seems inconsistent to conceptualise duties of justice without recourse to the nation state, but at the same time to fall back on the political instruments of the nation state in enforcing the fulfilment of these duties, in particular domestic tax law and tax administration as a collection apparatus supported by a monopoly on the use of force.

It is therefore unsurprising that cosmopolitan literature often advocates the ideal solution of at least partial supranationalisation or even globalisation of taxation itself. A call, for instance, for the establishment of an ‘International Tax Organisation’ is quite common, or for the transfer of taxing rights to existing international or supranational organisations such as the EU, OECD, WTO or UN.^61^

Apart from the enormous political obstacles that would have to be overcome in building the supranational institutions necessary for effective tax collection, the question arises as to whether the advocacy of moral cosmopolitanism requires such a structural transformation at all. In other words: does moral cosmopolitanism commit us to legal cosmopolitanism, especially with regard to taxation?^62^

The strongest arguments against such a commitment are pragmatic in nature. The advantages of specialisation and division of labour speak in favour of leaving the responsibility for the revenue side of a global redistributive mechanism at the state level.^63^ The ethical cosmopolitan, who does not see distributive duties as depending on certain types of co-operation or institutional participation, may, without running into contradictions, refer to the particularities of the state–citizen relationship for the fulfilment of these duties.^64^ Similarly, it is not contradictory in principle for the cosmopolitan to handle the expenditure side of a redistributive mechanism at the supranational level. The only result of this is the necessity of an intermediate transfer of resources from the receiving entity to the distributing entity.

### B. Global Redistribution Within the Existing Institutional Framework

If we ignore the possibility of a (partial) supranationalisation of taxing rights, either because it is regarded as politically unfeasible or because of doubts about effective enforceability, what options are available in order to achieve more distributive justice on a global scale within the current structural framework of international taxation?

Some ideas for how global redistribution could be achieved do not fall into the category of tax policy as strictly understood. The demand for more transparency is one of the most prominent proposals, especially in accounting for international trade in raw materials, in order to counteract the ‘siphoning’ of national resources by corruption and tax avoidance.^65^

The tax policy debate that deals with the ‘how to redistribute’^66^ dimension of international tax justice, in the sense of how the distributional outcomes the international framework produces could be made more favourable to developing countries, merits comprehensive treatment on its own and is beyond the scope of this article, so an overview here is necessarily sketchy. A key part of this debate revolves around options for strengthening the role of source in how taxing rights are allocated,^67^ and correspondingly the revenue share of developing countries that tend to be source countries.^68^ Policy options designed to strengthen source taxation include limitations on interest deductions and withholding taxes, coupled with an exemption of revenue subject to the withholding tax in the taxpayer’s state of residence, or with a matching tax credit corresponding to the amount of withholding tax.^69^

The structural reforms to the current system that are on the table currently as an outcome of the OECD’s BEPS project may initiate a paradigmatic shift in how taxing rights are allocated internationally.^70^ Although much depends on details that are yet to be clarified, the two-pillar approach might, if implemented, change the dynamics of international tax competition (due to global minimum taxation envisaged in Pillar 2).^71^ Pillar 1 of the current proposal would amount to a departure from the current structural paradigm in that it would introduce an element of (at least partial) formulary apportionment of taxing rights.^72^ The distributional issues may, under a reformed system, thus take on a different form and technical outfit—in principle, however, they remain the same. Distributional coordination as a politically challenging answer to ‘who should receive how much and why’ will survive any of the envisaged changes in how to go about such coordination.

## Distributive Justice, Moral Agency and Group Rights

This section returns to the initial question of whether a valid argument can be made that a (re)allocation of taxing rights at the expense of some states to the benefit of others is a *requirement of justice* that can be broken down into duties of justice of some states to fulfil corresponding rights of other states.^73^ As long as there is no reform of international taxation of the sort that implements legal cosmopolitanism by introducing global institutions with taxing rights of their own, taxing rights can—by definition—only be distributed among states.

A philosophical argument in favour of a requirement of justice to redistribute taxing rights among states can take two forms: it can be based either on an account of collective rights and responsibilities that qualifies states as proper moral agents capable of a relationship of justice on the collective level itself^74^ or on an instrumental argument that sees the redistribution of taxing rights as an adequate way of discharging distributive duties towards individuals. In the following sections, it will be argued that both strategies are unsuccessful.

### A. Justice as a Matter of Rights and Duties: Who Is Involved?

In the political context, justice is commonly conceptualised as a relational term, as an ideal quality of the relationship between moral agents.^75^ Since such relationships are often embedded in and formed by social institutions, understood as networks of interaction with a certain degree of consolidation, the justice predicate is often also applied to the design of these institutions themselves and the way individuals that are subject to them are treated.^76^

According to this understanding, the demands of justice determine the normative responsibilities we have to one another.^77^ A just social order, then, is one that is in line with and gives effect to these responsibilities. Rights and responsibilities are flip sides of the same moral coin: in distributive contexts, we look to rights to determine who is entitled to what, and to the corresponding duties to determine who bears the responsibility to honour these rights and protect the legitimate claims arising from them.^78^

A theory of justice that is able to provide guidance in political debates such as the one about content and scope of (re)distributive duties in international taxation must answer the following questions: what are the preconditions for moral agency and responsibility? Consequently, what types of entities can be bearers of moral duties? Whose rights do these duties correspond to? Who are the moral agents that matter on the beneficiary side? Are both sides linked in the sense that moral agency is the prerequisite for both rights and responsibilities alike?

Variations in the answers to these questions cut across the different groups of theories in the global justice debate that were presented above, which is why these issues are presented as a separate matter here. The structure of moral relationships has far-reaching implications for the debate about justice in international taxation as any claims that are made in terms of who owes what to whom must, to be convincing, be in line with who potentially is in a relationship of rights and duties of justice at all.

### B. Individual versus Collective Rights and Responsibilities

Most moral theory operates upon a traditional conception of individual agency that leads to individual rights and responsibilities. Rights are commonly conceptualised as protecting the choices or the well-being (or both) of individuals, with reference to their moral status and capability of moral agency.^79^

In the political context in general, however, and in the tax context in particular, we are rarely confronted with identifiable causal chains that link particular individuals to particular states of the world. When speaking about distributive justice within states and across state borders, we are dealing with large social collectives. However, the claims that we make regarding rights and duties of justice that individuals have towards each other cannot simply be transposed from the individual to the collective level.

Sometimes we thus encounter a ‘responsibility shortfall’:^80^ aggregating individual responsibilities does not cover the overall harm that has been caused, nor does it allow us to determine who has to remedy an unsatisfying situation, such as morally arbitrary inequality. A way of preventing such shortfalls is acknowledging some form of genuine collective responsibility that goes beyond an aggregation of the individual responsibilities of the collective’s members. The question that the discussion about collective responsibility has thus sought to answer is whether collectives themselves can be bearers of duties at all, whether we can justifiably hold groups *qua groups* responsible for remedying a situation that is morally unacceptable.^81^

#### (i) Collective responsibility

If we accept the idea of collective responsibility in principle, the implications we can draw from it depend on two issues: what criteria must social groups meet in order to qualify for some form of collective responsibility? And how, if at all, does collective responsibility translate into individual responsibility and vice versa?^82^

One influential account of collective responsibility is based upon the existence of a sufficiently robust mechanism of centralised decision making.^83^ The strategy here is to argue that the collective body in question is similar to an individual in the respects that are relevant for the ascription of moral agency. If moral agency and responsibility presuppose decision-making capacities of a certain kind, which include being able to choose from available alternatives and to follow through on such choices, we have a reason to treat collectives that exhibit these capacities to a sufficient degree as moral agents themselves. Collective decision making as such is a standard concept, but the question is whether and under what conditions it justifies an ascription of moral agency and responsibility.

Even if we can form a convincing account of collective responsibility, however, the following problem remains: a convincing account of collective responsibility alone would not suffice to establish duties of justice to redistribute taxing rights (or in fact any other kind of resource, generally speaking) on the *international* level. It would only establish that on the ‘giving’ side it could be states, as collectives, that bear a duty to redistribute.^84^ It would not suffice to establish that on the *recipient* side it would be other states, as collectives, that would have a claim of justice to be given a larger part of a particular tax cake. Showing that states and their governing bodies in which deprived individuals live are the correct recipients of efforts to discharge collective remedial responsibilities crucially depends on a collective conception of rights as well.

#### (ii) Group rights

The debate about which—if any—collective entities are proper candidates for bearers of rights has become known under the heading ‘group rights’. This debate has similarities to the one concerning collective responsibility, in that part of it is about the conditions that collective entities must meet in order to qualify as right holders, whatever the right may be. This is an ontological debate about the moral status of different types of groups that has paved the way for a ‘corporate conception’ of group rights. Here again, degrees of integration and institutionalised forms of collective decision making are among the candidates for features that justify treating them as moral agents capable of having rights *of their own*.^85^ Treating groups as right holders analogous to individuals has been criticised, however, as presupposing an ontological status of groups over and above that of their members that is hard to justify.^86^

In order to avoid these ontological problems, the ‘collective conception’ offers a different view of group rights as more than a simple aggregate of individual rights, but less than or different from rights that could only be borne by a collective with quasi-personal qualities. The most influential defence of the collective conception has been presented by Joseph Raz, who acknowledges group rights under the condition (among others) that they relate to an ‘interest of individuals as members of a group in a public good and the right is a right to that public good because it serves their interest as members of the group’.^87^ They differ from aggregations of individual rights in that ‘the interest of no single member of that group in that public good is sufficient by itself to justify holding another person to be subject to a duty’.^88^ As an example for such a collective right, Raz mentions the right to self-determination, which people are interested in as members of a group, and to which they would not have a sufficiently reasoned claim as individuals seen separately.^89^

Neither the corporate nor the collective conception of group rights can avoid a type of criticism that refers not to the contentious assumptions we would have to make about what kind of entities groups are, but to the negative consequences acknowledging group rights may have for the content and protection of individual rights. The argument here is that group rights may have the potential to oppress individual rights, both those of people who belong to the group in question and those of people who do not. It is difficult to see how not to fall into an aggregative framework, where the interests of the many may amount to a collectively held right that is in conflict with the individual rights of others that have less weight, particularly when group rights are understood along the lines of the collective conception. Resolving such tensions and conflicts in favour of group rights, and using them as reasons to infringe individual rights, is difficult to reconcile with a commitment to honour the ‘separateness of persons’.^90^ The oppressive potential is particularly acute in groups with involuntary membership, where a lack of realistic exit opportunities would hamper individuals’ options to take the protection of their individual rights into their own hands by leaving the group and joining another. There may be ways to deal with the problem of oppressive groups by adding to the list of qualifying criteria a requirement that groups must themselves respect certain rights of its individual members, such as a right of exit. With respect to citizenship, however, a mere right of exit does not suffice to establish voluntary membership in a political community; without the opportunity to gain citizenship in another state, the option of choosing no citizenship at all does little to alleviate the oppressive potential that group rights may have for the individual citizen. As a consequence, the set of groups that qualify under these conditions is narrowed considerably so that, at least in today’s political reality, many states would fall through the net, because their citizens cannot exercise their right of exit.

The problems discussed here do not support the conclusion that there are no collective moral rights of states *qua* states in international relations at all;^91^ besides limitations on the kind of states who qualify as right holders, limitations on the kind of rights that could be held by groups may also serve to reign in oppressive potential.^92^

### C. International Tax Justice as a Matter of Collective Rights and Responsibilities

Arguing that it would be states *per se* that have, as a matter of justice, a right to a certain share of taxing rights *vis-à-vis* other states requires a convincing conception of group rights that encompasses taxing rights as well.

An individual right to taxing rights does not make much sense in the first place, since taxation is not an individual, but a necessarily collective enterprise. We can make sense of individual rights to a certain share of socio-economic resources, of being granted access to the means of subsistence, and the revenue generated by a certain share of taxing rights borne by a political unit to which individuals belong typically has implications for the resources they have access to. The international tax justice debate, however, oscillates between the socio-economic rights of individuals and the collective duties of states to honour these rights with the help of reforms in international tax law.

Often, duties of distributive justice that exist directly *vis-à-vis* the recipient state as the relevant moral subject are simply presupposed. This, however, does not sit well with the methodological individualism of cosmopolitan theories. The intuitive force of cosmopolitan theories crucially depends on their core assumption that it is people’s status as individual human beings who are capable of moral agency that forms the basis for duties of distributive justice towards them and that calls for a reason to draw limits to such duties according to criteria such as membership of a political community are, from a moral standpoint, seemingly arbitrary. So, at least if cosmopolitanism’s appeal is invoked to justify a call for extending the moral perspective beyond national confines, holding that the relations between states themselves are governed by the same principles of distributive justice as between individuals is *prima facie* surprising. Interestingly, the cosmopolitan literature is divided and at times opaque about whether the monist claim that justice means the same and requires the same both nationally and internationally is a claim about the relationship between individuals or between groups—such as states—or some combination of both.

Missing in this debate is a connection between the individual and the collective level, a coherent argument as to how exactly the moral rights of individuals would give rise to a moral claim of their state towards another state regarding the substantive design of the international tax system.

Even if we set aside the objections that have been brought forward particularly against the collective conceptions of group rights, another obstacle remains on the way to treating taxing rights as one particular instance of such collective rights. This has to do with the content that group rights could have according to the Razian conception: the conditions under which a sharing of interests among individuals could be the basis for a genuine group right refer to those interests being directed at some form of public good, such as collective self-determination. The range of group rights that can be justified in this way is, according to Raz, from the beginning substantively limited to public goods. Seen from an international tax perspective, justifying a Razian group right to share the right to tax income and profit to a certain extent would require conceptualising taxing rights as a public good. Bearing in mind the standard criteria for public goods (being non-rivalrous and non-exclusive), taxing rights do not present themselves as a natural candidate. Assuming that multiple taxation is to be avoided, more extensive taxing rights of one political unit would diminish the taxing rights of other political units; seen globally, a right to tax particular kinds of income and profit is both a rivalrous and an exclusive good. But what about the internal perspective: can taxing rights be classified as public goods *within* the (national) collective that would hold them? This would depend on the kind of access that individual members of the group would have to such rights and on how they would benefit from an international rearrangement that leaves their group with more extensive taxing rights. Non-rivalrous and non-exclusive individual access to taxing rights themselves is hard to see at all, given the nature of taxation as a public capacity. In order to provide a link between taxing rights and group members, we must look to the fiscal consequences of those rights, to the revenue that would actually result from those rights and their extension by international rearrangement. With respect to these resources and the indirect sense in which group members would enjoy access to the taxing rights that have led to their availability, the familiar classification as public or private goods makes perfect sense. The focus then lies on the particular use of whatever additional revenue results from a rearrangement of taxing rights in the beneficiary state (if any, due to international tax competition). If the classification of taxing rights as public goods depends on the resulting revenue consequently being used to fund public goods to which the group’s members have non-rivalrous and non-exclusive access, the argument for a group’s right to enjoy more extensive taxing rights *vis-à-vis* another group survives only in stump form, if at all. Funding public goods is hardly the only use of tax revenue. Funding redistribution of resources within the group already blurs the picture, as access to the revenue thus used would fail the criterion of non-exclusivity.

Taken together, these obstacles call for a departure from the idea that rearranging taxing rights can be justified as a genuine duty of justice on the international level. Going back to the morally less problematic individual level, however, we would need an additional argument as to why duties of justice towards individuals are adequately discharged by a certain form or reform of international taxation as a collective enterprise.

### D. Interpersonal Tax Justice as a Matter of Individual Rights and Responsibilities

Could we bypass the group rights problem and still arrive at a duty of justice to redistribute taxing rights internationally, ie from one state to another? Without any contentious assumptions about the moral status of groups, instrumental reasons may speak for involving states as collective agents in a certain mechanism of resource allocation. In terms of practical organisation, this may simply be the most effective way of discharging duties of justice towards individuals. The instrumental argument, however, crucially depends on the assumption of a benevolent and capable government on the part of the recipient state that ‘passes on’ tax revenue to its constituents, either by direct transfer or by making use of it in a way that increases people’s well-being. But when it comes to compensating for resource inequality among individuals, the transfer of taxing rights to states and the governments responsible for ensuring their life chances does not make sense under all circumstances.^93^ The instrumental argument is valid only if rearranging international taxation is an effective way of improving those individuals’ life chances. It is therefore sufficient, but also necessary, to assume that international redistribution of taxing rights is an adequate mechanism in order to reduce resource inequality among individuals across the globe. The necessity of this assumption is problematic given that there is no readily available answer to the empirical question of how various modes of resource transfers across national boundaries compare in terms of effectively helping individuals in deprived circumstances. In principle, there are other ways in which taxation as a coercive state mechanism *vis-à-vis* individual citizens could be used to improve the life chances of individual non-citizens who would otherwise lack the resources needed to lead a minimally decent life, such as using tax revenue to finance foreign aid and development aid.^94^ The problem here, however, is that past experiences with many of these channels have been far from unambiguously positive.

This is not the place to give an adequate account of the controversial debate about the causes of global poverty and inequality, in particular the relationship between unfavourable starting conditions and political versus individual responsibilities, and to compare the ways of combating them.^95^ In any case, a quick glance at the countries in which an above-average number of people is affected by poverty makes it seem unrealistic to rely on governments as the most effective channels for redistributing resources.^96^ The instrumental argument, without a proper empirical backup, is therefore unconvincing.^97^ With a rearrangement of taxing rights, we thus appear to run into the same problems that have been well studied with respect to direct foreign aid in the form of lump-sum resource transfers. In terms of what is being transferred (financial resources) and who has the power to decide on how these resources are put to use, there seems not to be much of a difference between a direct aid transfer and the granting of an additional portion of taxing rights.^98^

Interestingly, cosmopolitan calls for rearrangements in the international tax arena at times acknowledge that they must rely on the benevolent government assumption, but at the same time treat it as an unproblematic detail: ‘Clearly massive reductions in existing human rights deficits could be achieved by allowing poor countries to collect reasonable taxes from MNCs and from their own most affluent nationals, *assuming the resulting revenues were appropriately spent*.’^99^

The upshot of these observations is that it is difficult to combine the structure of our moral relationships and the state of the world as it is, partitioned into states that cannot all simply be assumed to cater most effectively to their citizens’ well-being, into an argument for a reform of international taxation that leaves more taxing rights and thus more revenue to some states than others. Collective rights are difficult to defend, and without them we run into the problems of the instrumental argument.

Where does this leave us? The challenges that collective rights and responsibilities come with do not mean that the collective level cannot play any role in giving practical effect to moral obligations that reach across national boundaries, or that international taxation has no role to play in this endeavour. After all, taxing rights can only be distributed and redistributed on an international, and therefore collective, level. As a matter of political experience, the instrumental argument in favour of utilising the taxing rights channel for resource redistribution with a view to individuals as recipients may still hold in some—or even many—contexts. Still, from a theoretical perspective, the individual and collective levels must be distinguished: duties of distributive justice that hold between individuals cannot simply be assumed to hold between collectives or between collectives and individuals. Caution is required here, given the fact that even if it may be desirable that governments act as ‘trustees of humanity’,^100^ in reality such trusteeship often is not even directed towards their own citizens.

## Alternatives to Distributive Justice in International Taxation

The conclusion that genuinely *international* duties of distributive tax justice are difficult to construe does not, of course, mean that the international context is a realm in which no moral principles operate at all.^101^ The previous sections have dealt with the specific claim that affluent countries should agree to a rearrangement of taxing rights, particularly with respect to the profits of multinational enterprises, to the effect that a larger portion of the tax cake is left to less affluent states. If such rearrangements cannot be framed as genuine duties of justice, how else can we make sense of them, and what would be the difference compared to a rearrangement required by principles of distributive justice?

### A. Duties of Justice versus Humanitarian Obligations

In the global justice debate, one of the main issues is whether a requirement to redistribute has its roots in duties of humanitarian assistance to people in need, interpersonal duties to establish global distributive justice or obligations of good conduct and fairness between states as members of an international order based on principles of equal standing, fairness and co-operation.

From a tax point of view, we must ask ourselves: what difference does it make? After all, any type of redistribution or aid, no matter what it is called or how it is justified, must be financed.^102^ The first significant difference is of a quantitative nature: if, on the basis of a cosmopolitan theory of justice, the circle of those potentially obliged and entitled to redistribution does not end at national borders, the amount of resources that must be ‘fed in’ on the revenue side of the redistributive scheme increases dramatically.

Further differences relate to the specific content of what actions duties of justice require from us. Duties of distributive justice are more far-reaching than duties of humanitarian assistance, which refer to the global provision of a certain minimum socio-economic standard for all people and do not include a genuinely egalitarian component.^103^ In short, as soon as no one starves or freezes to death (and in keeping with what a generally accepted human rights catalogue would otherwise require), these duties relating to humanitarian assistance are fulfilled, no matter how many other people live in abundance.

In addition, genuine duties of justice are considered stronger^104^ or more robust than mere auxiliary duties in the sense that they justify third party sanctions in the event of non-compliance. Even if there is no third party competent to impose sanctions in the international context, the range of legitimate political sanctions can be extended by foreign policy measures.^105^

The qualification of a resource transfer as either the discharge of a distributive duty or humanitarian aid also has a symbolic-expressive component that should not be underestimated.^106^ Unlike the ‘arrogant generosity’ of voluntary support, the burden of justification in the case of unfulfilled distributive duties lies with those responsible for their fulfilment. A rhetorical asymmetry that can be observed in international tax policy fits this symbolic difference: while, from the point of view of economically stronger industrialised countries, resource transfers in the form of taxing rights or tax investment promotion are communicated as generous gifts, from the point of view of the beneficiary developing countries, it is a question of the fulfilment of international duties of justice that may well be demanded, not just met with gratitude.^107^

### B. Tax Cakes versus Whole Meals

Speaking of international tax cakes that should be distributed fairly may be shortsighted. When it comes to individuals’ well-being and life chances, what matters is not only or primarily the division of tax cakes of whatever size (which, in keeping with the nutritional metaphor, enter the revenue pool at a fairly late stage of the distributive game, as a type of dessert), but the whole meal that is the world economy and the gains it generates that are inevitably distributed somehow. This meal has many courses, shaped by many legal regimes: international trade arrangements, environmental treaties, the protection of intellectual property and so forth. All of these regimes affect the overall distributive outcome, which brings us back to the initial differentiation between whether and how to redistribute, ie between the reasons to redistribute resources globally at all and the specific instrumental ways to achieve the task.

Acknowledging that the international tax regime is only one of the many nuts and bolts of a whole body of rules that shape the distributive outcomes of the global economy prompts the question: if there is a bigger picture to be seen here, what does that mean for the ‘international tax justice’ debate? Is it an appropriate endeavour at all to come up with specific principles of distributive justice that should guide international tax policy?^108^ Or is the debate about international tax justice and what distributive principles would require in terms of institutional and substantive reform in international taxation more appropriately conceived as one subset of a larger ‘global trade justice’ debate?^109^

In light of the difficulties that accompany the instrumental argument for a redistribution of taxing rights, international taxation should not be overwhelmed with expectations as to its suitability for the task of compensating for global inequality and poverty. This is not to call into question the undisputed need to find a solution to these problems. While a comprehensive, globally coordinated reform might be the preferred scenario, there is little reason to hope for its political feasibility. Of all the other instruments that could be part of second-, third- and fourth-best strategies, international tax law is one. The discussion here was limited to problems of moral suitability from one particular angle; practical suitability and potential obstacles to an effective implementation of redistributive aims via a reform of the international tax system have not been the focus here. Giving due treatment to international taxation in a comprehensive global trade justice debate would require attention to both types of suitability.

## Conclusion

If we are willing to recognise considerations of justice—along with other criteria such as efficiency and administrability—as legitimate design principles for international tax policy, it is inevitable to reflect on the specific content of these principles of justice and to take account of controversial issues. Otherwise, theoretical considerations as well as their practical implications will remain vague, and for this reason alone run the risk of having little political and legal impact.

The previous sections have cast doubt on whether a coherent argument can be made that reliance on international taxation as a policy instrument in order to alleviate global inequality is a matter of *distributive justice*, thereby calling into question approaches that see international tax policy as faced with this task under this particular heading. This does not mean, of course, that any political decision or agreement to utilise tax policy in this way would be incoherent or necessarily ineffective. It also does not mean that arguments based on other accounts of justice could not succeed. In particular, the critical argument advanced here does not affect the claim that procedural justice requires strengthening the inclusivity of the international tax framework so as to give less affluent states a stronger voice at the bargaining table than they have had in the past.

The main argument of this article, however, is that although the theories of distributive justice presented here differ in terms of the overall redistribution of resources they require, none of them necessarily lead to distributive duties among states to reallocate taxing rights amongst one another. The differentiation between the interpersonal and the international level as regards the parties to a relationship governed by principles of distributive justice is not merely a terminological issue, but has significant implications for tax policy: considering the difficulties with construing genuinely collective rights of states with respect to taxing rights and absent a robust assumption of a benevolent and capable government on the recipient side, the reallocation of taxing rights from state to state does not necessarily help when it comes to fulfilling duties of justice towards individuals. The conceptual reasoning behind this conclusion is that distributive justice as it pertains to (international) tax policy is adequately understood as leading to interpersonal relations of claims and duties.

## Footnotes

1 On the various dimensions of distributive consequences, see Ivan O Ozai, ‘Two Accounts of International Tax Justice’ (2020) 33 Canadian Journal of Law & Jurisprudence 317, 322–7; note that this issue is not the same as the ‘fair share’ problem that developed countries address when complaining about unfairness in the international taxation particularly of multinational enterprises whose business models require little physical presence and thus lead to little taxing rights of market countries under the current system. Although this issue is framed in terms of fairness and justice, it is not the focus of this article; for a short overview of this dimension of the tax justice debate, see Michael P Devereux and others, *Taxing Profit in a Global Economy* (OUP 2021) 6–8.

2 The Musgraves’ seminal work on ‘inter-nation equity’ can be interpreted as involving such a claim, see Richard A Musgrave and Peggy B Musgrave, ‘Inter-Nation Equity’ in Richard M Bird and John G Head (eds), *Modern Fiscal Issues: Essays in Honor of Carl S Shoup* (University of Toronto Press 1972) 74; see also Nancy H Kaufman, ‘Fairness and the Taxation of International Income’ (1997) 29 Law & Pol Int Bus 145, 203; Reuven S Avi-Yonah, ‘Globalization, Tax Competition, and the Fiscal Crisis of the Welfare State’ [2000] Harv L Rev 1573, 1650: ‘when a choice is presented between two otherwise comparable alternative rules, one of which has progressive and the other regressive implications for the division of the international tax base between poorer and richer countries, the progressive rule should be explicitly preferred to the regressive one. In the absence of a world taxing authority that can redistribute tax revenues directly, and given the paucity of foreign aid from developed to developing countries, such a concept of inter-nation equity has the best chance of achieving meaningful distributive goals’; Ilan Benshalom, ‘The New Poor at Our Gates: Global Justice Implications for International Trade and Tax Law’ (2010) 85 NYU L Rev 1, 70: ‘a redistributive allocation of taxing rights seems to be a relatively effective way to fulfill relational-distributive obligations’; Ozai (n 1) 327, 338–9; Tarcísio D Magalhaes and Ivan Ozai, ‘A Different Unified Approach to Global Tax Policy’ (2021) 4 Nordic Journal on Law and Society 18.

3 Liam Murphy and Thomas Nagel, *The Myth of Ownership: Taxes and Justice* (OUP 2002) 76 ff; Benshalom, ‘The New Poor at Our Gates’ (n 2) 1.

4 Murphy and Nagel (n 3) 41 expressly exclude the international/global dimension from their analysis, yet acknowledge the need for further research; recent exceptions are Tsilly Dagan, *International Tax Policy: Between Competition and Cooperation* (CUP 2017) ch 6, 7; Gillian Brock, ‘Taxation and Global Justice: Closing the Gap between Theory and Practice’ (2008) 39 Journal of Social Philosophy 161; Gillian Brock and Thomas Pogge, ‘Global Tax Justice and Global Justice’ (2014) 1 Moral Philosophy and Politics 1; Ilan Benshalom, ‘How to Redistribute?: A Critical Examination of Mechanisms to Promote Global Wealth Redistribution’ (2014) 64 UTLJ 317; Thomas W Pogge and Krishen Mehta (eds), *Global Tax Fairness* (OUP 2016); Miranda Stewart, ‘Redistribution Between Rich and Poor Countries’ (2018) 72 Bulletin for International Taxation 297.

5 Given the multifaceted nature of the debate about justice in international taxation, a caveat is in order: this article does not aim to give a comprehensive account of all types of arguments that have been made about the proper understanding of justice in international taxation, the role justice should play in tax policy and the specific options by which implementation could feasibly achieved. As a result of this limitation, the criticisms advanced here do not affect the validity of other arguments that have been brought forward in support of a greater role for justice in international taxation. This is true particularly with respect to accounts of how and why the status quo in international taxation must be considered as unjust that do not refer to distributive duties to reallocate taxing rights between states. A complete taxonomy of justice considerations would certainly include procedural and corrective accounts. A comprehensive treatment of the issue must also include an analysis of international tax competition and the fairness problems caused by a tilted ‘playing field’, and of the role that international institutions such as the OECD and the WHO play in shaping international tax policy according to the interests of those with the most influence and power at their drafting and bargaining tables.

6 The term ‘taxing rights’ is used here as a legal term and does not refer to any kind of moral right.

7 Michael Blake and Patrick T Smith, ‘International Distributive Justice’, *The Stanford Encyclopedia of Philosophy* (Summer edn, 2021) s 1 <https://plato.stanford.edu/archives/sum2021/entries/international-justice/> accessed 2 July 2021: ‘in a world as connected as our own, is it justifiable that some have so much while others have so little?’

8 Benshalom, ‘The New Poor at Our Gates’ (n 2) 37.

9 This distinction has been first introduced and more comprehensively discussed (in German) in Johanna Stark, ‘Verteilungsgerechtigkeit als Prinzip des Internationalen Steuerrechts’ [2019] Steuer und Wirtschaft 71, 72–4.

10 Itai Grinberg, ‘Comment on Stewart’s “Redistribution Between Rich and Poor Countries”’ (2018) 72 Bulletin for International Taxation 310, 311; Peter Hongler, *Justice in International Tax Law: A Normative Review of the International Tax Regime* (IBFD 2019) 11.

11 Charles R Beitz, ‘Does Global Inequality Matter?’ (2001) 32 Metaphilosophy 95, 110.

12 For an overview, see David Miller, ‘Justice’, *The Stanford Encyclopedia of Philosophy* (Fall edn, 2017) s 2.2 <https://plato.stanford.edu/archives/fall2017/entries/justice/> accessed 2 July 2021.

13 Benshalom, ‘How to Redistribute?’ (n 4) 317 stresses the need for a separation between ‘why’ and ‘how’ of international redistribution.

14 The instrumental dimension would also matter in an argument from impossibility, ie when arguing that because altering the current international tax system so as to achieve more distributive justice cannot be done (for reasons to be included in the argument), there is not much use in debating about *why* it should be done. This is not, however, the type of argument advanced here.

15 Except for a brief overview in s 4B.

16 Kaufman (n 2) 202.

17 Nor is there much help to be expected from the criteria developed for the revenue side, such as the ability to pay principle; see Wolfgang Schön, ‘International Tax Coordination for a Second-Best World (Part II)’ [2010] World Tax Journal 65, 73: ‘Ability to pay helps to define the cake, but it does not help to slice it.’

18
*OECD*, ‘Action 8–10 Final Report 2015: Aligning Transfer Pricing Outcomes with Value Creation, 2015’ <https://doi.org/10.1787/9789264241244-en> accessed 2 July 2021; Susan C Morse, ‘Value Creation: A Standard in Search of a Process’ (2018) 72 Bulletin for International Taxation 196.

19 Some regard this path as not promising anyway, since the distribution to be made is a political decision that must be taken by negotiation and cannot be derived from a ‘correct’ understanding of value creation: Schön (n 17) 93: ‘one should not look for the holy grail of a “natural” allocation of taxing rights’; similarly Morse (n 18) 199: ‘The premise of the forum is that its task is a search for compromise rather than a search for truth.’

20 See Johanna Hey, ‘“Taxation Where Value is Created” and the OECD/G20 Base Erosion and Profit Shifting Initiative’ (2018) 72 Bulletin for International Taxation 203.

21 ibid 204.

22 On the ‘international tax cake’, see Schön (n 17) 73.

23 For a more elaborate exposition of the global justice debate in the context of international taxation, see Stark (n 9) 74–8.

24 This question is typically discussed under the heading ‘inter-individual vs internation equity’, see Diane Ring, ‘Democracy, Sovereignty and Tax Competition: The Role of Tax Sovereignty in Shaping Tax Cooperation’ (2009) 9 Florida Tax Review 555.

25 This is even clearer in the case of the term ‘internation equity’, which is familiar from tax discourse and was coined by Musgrave and Musgrave (n 2).

26 Christian Barry, ‘Global Justice: Aims, Arrangements, and Responsibilities’ in Toni Erskine (ed), *Can Institutions Have Responsibilities? Collective Moral Agency and International Relations* (Palgrave Macmillan 2003) 224: ‘Cosmopolitan views take individual persons as their subjects, and focus directly on the distribution of goods and ills (eg wealth, education, health, poverty, illiteracy, and morbidity) amongst them.’

27 Justice is sometimes defined as the absence of arbitrary inequality, cf Miller, ‘Justice’ (n 3912) s 2.4.

28 One of the key representatives of this approach is Harry G Frankfurt, *On Inequality* (Princeton UP 2015); for representatives of such ‘sufficiency principles’, a state of affairs is unfair not because some people have more without justification than others, but because some do not have enough by a certain standard.

29 For a more comprehensive, encyclopaedic treatment of other conceptual nuances, see Deen K Chatterjee, *Encyclopedia of Global Justice* (Springer Science & Business Media 2011).

30 John Rawls, *A Theory of Justice* (rev edn, Harvard UP 2009).

31 ibid 81.

32 ibid 3.

33 Thomas W Pogge, *Realizing Rawls* (Cornell UP 1989); Charles R Beitz, *Political Theory and International Relations* (Princeton UP 1979); Thomas W Pogge, ‘World Poverty and Human Rights: Cosmopolitan Responsibilities and Reforms’ (Polity Press 2002); Charles Beitz, ‘Cosmopolitanism and Global Justice’ (2005) 9 Journal of Ethics 11.

34 Pogge, *Realizing Rawls* (n 33) 247.

35 Ayelet Shachar, *The Birthright Lottery: Citizenship and Global Inequality* (Harvard UP 2009) 2.

36 Blake and Smith (n 7) s 1.

37 Benshalom, ‘The New Poor at Our Gates’ (n 2) 37; Andrea Sangiovanni, ‘Global Justice, Reciprocity, and the State’ (2007) 35 Philosophy & Public Affairs 3.

38 Beitz, *Political Theory and International Relations* (n 33) 351.

39 Blake and Smith (n 7) s 3.

40 He further explained his position, which had already been set out in *A Theory of Justice* (n 30), in John Rawls, *The Law of Peoples* (Harvard UP 2001).

41 David Miller, *National Responsibility and Global Justice* (OUP 2007) ch 5; Michael J Graetz, ‘The David R Tillinghast Lecture Taxing International Income: Inadequate Principles, Outdated Concepts, and Unsatisfactory Policies’ (2000) 54 Tax Law Review 261, 277.

42 David Miller, ‘Cosmopolitanism: A Critique’ (2002) 5 Critical Review of International Social and Political Philosophy 80, 82: ‘slid[ing] from saying that every human being has equal moral worth to saying that therefore we are required to treat all human beings equally, in the sense that we have the same duties to each … is simply a non sequitur’.

43 Joshua Cohen and Charles Sabel, ‘Extra Rempublicam Nulla Justitia?’ (2006) 34 Philosophy & Public Affairs 147, 158f.

44 Thomas Nagel, ‘The Problem of Global Justice’ (2005) 33 Philosophy & Public Affairs 113.

45 ibid 115.

46 ibid 120; a similar argument is presented by Ronald Dworkin, *Sovereign Virtue: The Theory and Practice of Equality* (Harvard UP 2000) 6.

47 Brian Barry, ‘Humanity and Justice in Global Perspective’ (1982) 24 Nomos 219, 233 eloquently puts it as ‘trading partners vs fellow citizens’; Nagel (n 44) 138.

48 Nagel (n 44) 121.

49 ibid 119.

50 This section draws on an earlier study of how international tax law could be used as an instrument for redistributing resources globally, see Stark (n 9) 78–81.

51 Stewart (n 4) 306.

52 Thomas W Pogge, ‘An Egalitarian Law of Peoples’ (1994) 23 Philosophy & Public Affairs 195; Pogge, ‘World Poverty and Human Rights’ (n 33) 66–7; Thomas W Pogge, ‘Allowing the Poor to Share the Earth’ (2011) 8 Journal of Moral Philosophy 335.

53 This idea is complemented by Pogge’s criticism of the ‘international commodity privilege’, which enables members of government—legitimate and illegitimate alike—to enrich themselves with the help of a country’s natural resources, for example by selling mining rights to mineral resources. Pogge argues that this incentive structure contributes decisively to unstable political conditions, especially in resource-rich developing countries, also known as the ‘resource curse’, see Leif Wenar, ‘Property Rights and the Resource Curse’ (2008) 36 Philosophy & Public Affairs 2; similarly, Pogge criticises the ‘international borrowing privilege’, which makes it possible for illegitimate governments to enter into long-term payment obligations.

54 Thomas W Pogge, ‘The Health Impact Fund and Its Justification by Appeal to Human Rights’ (2009) 40 Journal of Social Philosophy 542.

55 Brock (n 4) 172.

56 Shachar (n 35) 15.

57 Brock (n 4) 171.

58 ibid; Gabriel Wollner, ‘Justice in Finance: The Normative Case for an International Financial Transaction Tax’ (2014) 22 Journal of Political Philosophy 458.

59 Brock (n 4) 172–4.

60 The administrative responsibility for tax collection does not necessarily have to go hand in hand with a competence to decide on its further use.

61 Brock (n 4) 176.

62 Pogge also points out that a distinction must be made between these two positions: Thomas W Pogge, ‘Cosmopolitanism and Sovereignty’ (1992) 103 Ethics 48, 49.

63 A further problem is the lack of morally relevant information on a supranational level; Alexander W Cappelen, ‘The Moral Rationale for International Fiscal Law’ (2001) 15 Ethics & International Affairs 97, 110; Stewart (n 4) 302: ‘The contemporary “tax state” developed over the last two centuries has a capability unique in recorded history to manage mass redistribution from rich to poor.’

64 Cappelen (n 63) 108 sees ‘state borders as convenient ways of allocating responsibilities that themselves derive from universal principles’; Robert E Goodin, ‘What Is So Special about Our Fellow Countrymen?’ (1988) 98 Ethics 663, 681 sees the citizen–state relationship with its specific duties as the best way of fulfilling the ‘general duties that everyone has towards everyone else worldwide’; see also Robert E Goodin, *Utilitarianism as a Public Philosophy* (CUP 1995) 282.

65 Brock and Pogge (n 4) 7; Allison Christians, ‘How Nations Share’ (2012) 87 Ind LJ 1407 presents arguments for more transparency with regard to the conclusion and outcome of international tax disputes, especially in the area of soft law.

66 Benshalom, ‘How to Redistribute?’ (n 4).

67 The two most relevant model conventions that serve as the basis for tax treaties differ in precisely this respect: compared to the OECD model convention, the UN model convention provides for a far greater extent of source taxation; cf United Nations Model Double Taxation Convention between Developed and Developing Countries 2011, Introduction No 3.

68 Among the proposals is a differentiated allocation of taxing rights depending on the parties involved: application of the residence principle between OECD countries could be paired with an application of the source principle when the distributional setting involves developed *vis-à-vis* developing countries; other proposals argue for exclusive taxing rights regarding certain types of revenue, such as interest on imported capital in developing countries, see Benshalom, ‘The New Poor at Our Gates’ (n 2) 37, 77.

69 Matthias Valta, *Das Internationale Steuerrecht zwischen Effizienz, Gerechtigkeit und Entwicklungshilfe* (Mohr Siebeck 2014) 598; Pasquale Pistone, ‘Tax Treaties with Developing Countries: A Plea for New Allocation Rules and a Combined Legal and Economic Approach’ in Michael Lang and Pasquale Pistone (eds), *Tax Treaties: Building Bridges Between Law and Economics* (2010) 440; for an overview, see Peter Mullins, ‘International Taxation and Developing Countries’ (2020) CGD Policy Paper 166 <www.cgdev.org/publication/international-taxation-and-developing-countries> accessed 2 July 2021.

70 OECD, ‘Statement by the OECD/G20 Inclusive Framework on BEPS on the Two-Pillar Approach to Address the Tax Challenges Arising from the Digitalisation of the Economy’ (January 2020) <www.oecd.org/tax/beps/statement-by-the-oecd-g20-inclusive-framework-on-beps-january-2020.pdf> accessed 2 July 2021.

71 OECD, ‘Tax Challenges Arising from Digitalisation—Report on Pillar Two Blueprint’ (October 2020) <https://doi.org/10.1787/abb4c3d1-en> accessed 2 July 2021; for comments on Pillar 2, see Michael P Devereux and others, ‘The OECD Global Anti-Base Erosion (“GloBE”) Proposal’ (January 2020) <https://oxfordtax.sbs.ox.ac.uk/files/oecdglobeproposalreportpdf> accessed 2 July 2021.

72 OECD, ‘Tax Challenges Arising from Digitalisation—Report on Pillar One Blueprint’ (October 2020) < https://doi.org/10.1787/beba0634-en> accessed 2 July 2021.

73 As Miller, *National Responsibility and Global Justice* (n 41) 232 has pointed out, there is ‘a large normative gap between identifying a state of affairs as intolerable and identifying agents, individual or collective, who have a responsibility to remedy it’.

74 Miriam Ronzoni and Laura Valentini, ‘Global Justice and the Role of the State’ in Thom Brooks (ed), *The Oxford Handbook of Global Justice* (OUP 2020) provide an analysis of how the state is conceptualised, by some, as a moral agent in the global justice debate and beyond.

75 On other dimensions of meaning, such as justice as a personal virtue in the Aristotelian tradition, see Miller, ‘Justice’ (n 12).

76 John Rawls has characterised justice as the first virtue of institutions; Rawls, *A Theory of Justice* (n 11) 110, describing social justice as being about the allocation of the benefits and burdens of social life.

77 Thomas Scanlon, *What We Owe to Each Other* (Belknap Press 2000).

78 Alexa Zellentin, Clare Heyward and Lucas Meyer, ‘Introduction: Responsibility in International Political Philosophy’ (2012) 19 Ethical Perspectives 613, 614; Joseph Raz, *The Morality of Freedom* (Clarendon Press 1986) 208: ‘A right is a ground for a duty of another. And a duty exists only if it would defeat certain conflicting considerations were they to exist.’

79 Adina Preda, ‘Group Rights and Shared Interests’ (2013) 61 Political Studies 250, 252.

80 Anna Stilz, ‘Collective Responsibility and the State’ (2011) 19 Journal of Political Philosophy 190, 193.

81 For this kind of remedial responsibility as opposed to outcome responsibility that is based upon some causal involvement in bringing about the morally unacceptable situation, see Miller, *National Responsibility and Global Justice* (n 41) 77.

82 This question is beyond the scope of this article, but is dealt with at length by Marion Smiley, ‘Collective Responsibility’, *The Stanford Encyclopedia of Philosophy* (Summer edn, 2017) s 4 <https://plato.stanford.edu/archives/sum2017/entries/collective-responsibility/> accessed 2 July 2021.

83 Philip Pettit, ‘Responsibility Incorporated’ (2007) 117 Ethics 171; Nagel (n 44) 141 refers to collective agency, collective engagement that can trigger collective responsibility, if a threshold of institutionalisation (eg by centralised decision making) is met.

84 Individual responsibilities to redistribute do not make much sense when we are talking about taxing rights.

85 Peter Jones, ‘Group Rights’, *The Stanford Encyclopedia of Philosophy* (Summer edn, 2016) s 4 <https://plato.stanford.edu/archives/sum2016/entries/rights-group/> accessed 2 July 2021.

86 For a comprehensive treatment of group agency and responsibility, see Christian List and Philip Pettit, *Group Agency: The Possibility, Design, and Status of Corporate Agents* (OUP 2011); against basing an account of group rights on an analogy to individuals Jan Narveson, ‘Collective Rights?’ (1991) 4 Canadian Journal of Law & Jurisprudence 329, 334: ‘’Only individuals can make decisions, can literally have values, literally engage in reasoning and deliberation’; George W Rainbolt, *The Concept of Rights* (Springer 2015) 208.

87 Raz (n 78) 208.

88 ibid.

89 ibid 208–9.

90 Jones (n 85) s 7: ‘In contemporary philosophy, rights-thinking is commonly opposed to maximizing consequentialism, of which utilitarianism is the prime example. Ascribing rights to individuals is a way of acknowledging their separateness as persons and of safeguarding them from the aggregative excesses of utilitarianism. But if the aggregation of interests can yield collective rights, a morality of rights may begin to assume the character of, and to present the same dangers as, the very sort of utilitarian thinking that many rights-theorists intend it to preclude.’

91 In international relations theory, ‘realist’ approaches emphasise power dynamics among states and and dominant motivations to pursue own interests, whereas ‘international society’ and ‘idealist’ theorists see more room for moral principles such as justice operating in the international arena; for an overview, see Catherine Lu, ‘World Government’, *The Stanford Encyclopedia of Philosophy* (Spring edn, 2021) s 2.1 <https://plato.stanford.edu/archives/spr2021/entries/world-government/> accessed 2 July 2021; Robert Schuett and Peter MR Stirk (eds), *The Concept of the State in International Relations: Philosophy, Sovereignty and Cosmopolitanism* (Edinburgh UP 2015); for a more detailed discussion of realist approaches, see WJ Korab-Karpowicz, ‘Political Realism in International Relations’, *The Stanford Encyclopedia of Philosophy* (Summer edn, 2018) <https://plato.stanford.edu/archives/sum2018/entries/realism-intl-relations/> accessed 2 July 2021; interestingly, part of the debate about justice in international relations focuses on whether states have a duty to obey international law; within this debate, the relations between states are more commonly construed according to procedural accounts of justice, see Hilary Charlesworth, ‘International Law and International Justice’ in Chris Brown and Robyn Eckersley (eds), *The Oxford Handbook of International Political Theory* (OUP 2018).

92 For more on these substantial limitations, see s 5C below.

93 Stewart (n 4) 306–7 discusses the tension between the goal of improving the situation of individual people and the fact that ‘the legal and political mechanisms of taxation are state-based’.

94 Miller, *National Responsibility and Global Justice* (n 41) 265–6.

95 Jeffrey Sachs, *The End of Poverty: How We Can Make It Happen in Our Lifetime* (Penguin 2005) gives an overview from an economic perspective; on the problems associated with traditional development aid, see William Easterly, *The White Man’s Burden: Why the West’s Efforts to Aid the Rest Have Done So Much Ill and So Little Good* (OUP 2006); the contentiousness of this matter is also taken up by World Bank, *Assessing Aid: What Works, What Doesn’t, and Why* (A World Bank Policy Research Report, OUP 1998).

96 Avi-Yonah (n 26) 222: ‘Corrupt governments often remain in power because of financial and military assistance from abroad, which enables them to ignore and suppress dissent within their borders.’

97 Miller, *National Responsibility and Global Justice* (n 10) 311–12.

98 See, however, Mitchell Kane, ‘Tax and Human Rights: The Moral Valence of Entitlements to Tax, Sovereignty, and Collectives’ in Philip Alston and Nikki Reisch (eds), *Tax, Inequality, and Human Rights* (OUP 2019) 100: ‘There is a massive difference between developed countries redirecting a portion of existing revenue toward the redress of human rights shortfalls in developing countries on the one hand, and a restructuring of the basic understanding of international tax entitlements such that developing countries have a superior claim to revenues as their own in the first instance on the other.’ The argument put forward here, however, is narrower: the structural similarity holds only with respect to the problem of securing adequate use of additional funds in countries faced with human rights problems.

99 Thomas W Pogge and Krishen Mehta, ‘Introduction’ in Thomas W Pogge and Krishen Mehta (eds), *Global Tax Fairness* (OUP 2016) 43.

100 Eyal Benvenisti, ‘Sovereigns as Trustees of Humanity: On the Accountability of States to Foreign Stakeholders’ (2013) 107 AJIL 295, 295–333.

101 See also n 91 above with further references on the realism/idealism debate in international relations theory.

102 Daniel Halliday, ‘Justice and Taxation’ (2013) 8 Philosophy Compass 1111, 1118.

103 Kok-Chor Tan, *Justice without Borders: Cosmopolitanism, Nationalism, and Patriotism* (CUP 2004) 21: ‘Duties of global justice would be, thus, more encompassing and would operate at a more fundamental level than what we may call duties of humanitarian assistance.’ On the difference between a ‘global minimum’ and an egalitarian motivation for introducing redistributive schemes on a global scale, see also Miller, *National Responsibility and Global Justice* (n 44) 119: ‘Humanitarian duties hold in virtue of the absolute rather than the relative level of need of the people we are in a position to help. Justice, by contrast, is concerned with the relations between the conditions of different classes of people, and the causes of inequality between them.’

104 Miller, *National Responsibility and Global Justice* (n 41) 249 regards humanitarian duties as having ‘lesser weight’.

105 ibid 248, 258, 261.

106 Halliday (n 106) 1118: ‘redistribution through coercive taxation has an important expressive function’.

107 Valta (n 69) 6.

108 For isolationist approaches, see Aaron James, ‘A Theory of Fairness in Trade’ (2014) 1 Moral Philosophy and Politics 177, 179; Aaron James, *Fairness in Practice: A Social Contract For a Global Economy* (OUP 2013) 123–5. James points out the benefit that such approaches are able to offer practically applicable principles and clear normative guidance in specific contexts; see also Mathias Risse, *On Global Justice* (Princeton UP 2012) 275.

109 Integrationist approaches are advanced, among others, by Simon Caney, ‘Just Emissions’ (2012) 40 Philosophy & Public Affairs 255 with respect to problems of justice related to climate change; Andrew Walton, ‘Trade Justice: An Argument for Integrationist, not Internal, Principles’ (2020) 28 Journal of Political Philosophy 51; with a similar impetus, Adam Kern, ‘Illusions of Justice in International Taxation’ (2020) 48 Philosophy & Public Affairs 151, 40 argues that ‘we should evaluate international tax regimes based on how well they bring about relatively abstract distributive patterns’.

